# The Role of Neuroimaging and Genetic Analysis in the Diagnosis of Children With Cerebral Palsy

**DOI:** 10.3389/fneur.2020.628075

**Published:** 2021-02-09

**Authors:** Veronka Horber, Ute Grasshoff, Elodie Sellier, Catherine Arnaud, Ingeborg Krägeloh-Mann, Kate Himmelmann

**Affiliations:** ^1^Department of Paediatric Neurology, University Children's Hospital, Tübingen, Germany; ^2^Institute of Medical Genetics and Applied Genomics, University Hospital, Tübingen, Germany; ^3^Grenoble Alpes University, CNRS, Grenoble INP, CHU Grenoble Alpes, TIMC-IMAG, Grenoble, France; ^4^Registre des Handicaps de l'Enfant et Observatoire Périnatal, Grenoble, France; ^5^CERPOP, SPHERE Team, University of Toulouse, Inserm, UPS, Toulouse, France; ^6^Clinical Epidemiology Unit, Toulouse University Hospital, Toulouse, France; ^7^Department of Pediatrics, Clinical Sciences, Sahlgrenska Academy, University of Gothenburg, Gothenburg, Sweden

**Keywords:** diagnosis, classification, genetic analysis, cerebral palsy, magnetic resonance imaging

## Abstract

Cerebral magnetic resonance imaging (MRI) is considered an important tool in the assessment of a child with cerebral palsy (CP), as it is abnormal in more than 80% of children with CP, disclosing the pathogenic pattern responsible for the neurological condition. MRI, therefore, is recommended as the first diagnostic step after medical history taking and neurological examination. With the advances in genetic diagnostics, the genetic contribution to CP is increasingly discussed, and the question arises about the role of genetic testing in the diagnosis of cerebral palsy. The paper gives an overview on genetic findings reported in CP, which are discussed with respect to the underlying brain pathology according to neuroimaging findings. Surveillance of Cerebral Palsy in Europe (SCPE) classifies neuroimaging findings in CP into five categories, which help to stratify decisions concerning genetic testing. Predominant white and gray matter injuries are by far predominant (accounting for around 50 and 20% of the findings). They are considered to be acquired. Here, predisposing genetic factors may play a role to increase vulnerability (and should especially be considered, when family history is positive and/or causative external factors are missing). In maldevelopments and normal findings (around 11% each), monogenic causes are more likely, and thus, genetic testing is clearly recommended. In the miscellaneous category, the precise nature of the MRI finding has to be considered as it could indicate a genetic origin.

## Introduction

Cerebral palsies (CP) cover a group of diseases characterized by common clinical characteristics: CP is permanent, but not unchanging; it involves a disorder of movement and/or posture and of motor function; it is due to a non-progressive interference/lesion/abnormality; this interference/lesion/abnormality is in the developing/immature brain (1). Thus, it includes a number of conditions of different etiologies. The diagnosis of cerebral palsy is based on phenomenology, not on etiology (2).

Neuroimaging is not part of the diagnostic criteria, but it helps to understand the etiology or at least the pathogenesis of the underlying brain disorder. Magnetic resonance imaging (MRI) is abnormal in more than 80% of children with CP, disclosing the pathogenic pattern responsible for the neurological condition (2, 3). There is consensus on an international basis that cerebral MRI is important in the assessment of a child with cerebral palsy (4). MRI is recommended as the first diagnostic step after medical history taking and neurological examination (5). With the development of comprehensive genetic diagnostics, the genetic contribution to CP is increasingly discussed (6), and the question arises about the role of genetic testing in the diagnosis of cerebral palsy.

In a recent review, inconsistency of phenotypic definition of CP used by studies that investigated genetic causes of CP has been highlighted, which limits the quality and interpretation of study findings (7). The use of Surveillance of Cerebral Palsy in Europe (SCPE) guidelines (1) has been recommended for a precise diagnosis and classification.

We would like to discuss the role of genetic testing in relation to CP as phenotypically defined by SCPE and with respect to neuroimaging findings in children with CP. For classification of the latter, we also refer to guidelines established by the SCPE as described below.

The above given definition of SCPE excludes progressive disorders or non-cerebral diseases leading to a loss of motor function and underlines that the diagnosis of CP should be confirmed at around the age of 4 years. Subtypes of CP are defined according to clear neurological traits and recorded as spastic (unilateral and bilateral), dyskinetic, or ataxic CP. Furthermore, the SCPE network developed several tools including hierarchical trees (decision and classification trees), a standardized data collection form, and a Reference and Training Manual (1, 8, 9). For a full description, go to https://eu-rd-platform.jrc.ec.europa.eu/_en.

### MRI Patterns—Distribution

The MRI classification system (MRICS) was developed and validated by SCPE, including a literature review and comparison to other classification systems (10). MRICS identifies typical MRI patterns in children with CP, associated with a specific timing of the brain compromise:

- *maldevelopments (category A)*, which originate in the first and partly in the second trimester of pregnancy.
- *predominant white matter injury (category B)*, which typically arises in the early third trimester of pregnancy and especially characterize brain lesions of preterm born children with CP.
- *predominant gray matter injury (category C)* defines lesions arising in the late third trimester or around birth of a term or near to term born child with CP.
- findings not corresponding to one of the three categories are classified as *miscellaneous (category D)*.
- *normal findings (category E)*.

In a first analysis of MRI patterns in children with CP representative of the population, maldevelopments made up for around 11% of cases, predominant white matter injury accounted for 49% of cases, and predominant gray matter injury for 21%, whereas miscellaneous findings were reported in 8.5% and normal findings in another 10.5% of the cases (11). MRI patterns of children with unilateral spastic, bilateral spastic, and dyskinetic CP were mainly lesional (77, 71, and 59%), while children with ataxic CP had more maldevelopments, miscellaneous, and normal findings (together nearly 80%). The latter groups were also more frequent in term than in preterm born with CP (nearly 40% vs. 22 and 13% in children born with a gestational age of 32–36 weeks and <32 weeks).

These patterns, characterizing brain pathology in CP, may serve as guidance when discussing the contribution of genetic testing to the diagnosis of CP. In maldevelopments, monogenic causes are very likely to play a role (12, 13), which is probably also the case for normal findings. Predominant white and gray matter injuries are considered to be acquired, but predisposing genetic factors may increase vulnerability (14, 15). The group of miscellaneous findings has to be studied in more detail with the question whether findings indicate a genetic disease.

With this background, we will discuss the current evidence for genetic findings reported in CP with respect to the underlying brain pathology.

### Genetic Testing in Cerebral Palsy

The presence of congenital anomalies in children with CP, the higher rates of CP in monozygotic than in dizygotic twins, and the higher risk of CP in consanguineous families compared with non-consanguineous first induced the discussion on a genetic background in the etiology of CP (16). A record-linkage study in three European regions with CP and congenital anomaly (CA) registers reported a higher prevalence of congenital anomalies among the CP population than in the general population. Prevalence was highest in children with ataxic CP (41.7%) and lowest in those with dyskinetic CP (2.1%); it was higher also among children born at term (13%) than among those born preterm (3.8%) (17). This indicates that genetic causes are more likely in the rare ataxic CP subtype and also more frequent in term than in preterm born children.

Increasing availability of comprehensive genetic testing such as microarray platforms and next-generation sequencing then has shed light on various genetic aspects linked to CP (15).

#### Genetic Testing Relevant for Individual Diagnosis

Here two different genetic categories are particularly relevant, copy number variations (CNVs), which can be assigned to a corresponding clinical phenotype, and sequence alterations in known CP-associated disease genes. The detection of mere genetic associations and vulnerability factors hardly plays a role in diagnostics as they are usually not robust enough.

- **Copy number variants (CNVs)** are understood as genomic deletions or duplications in copy number of genes. A minimum limit of CNV size of 50–100 kb is usually defined in the diagnostic setting. In a research environment and in special diagnostic cases, even significantly smaller CNVs can provide valuable information. Up to now, test methods of choice for detecting CNVs are mostly microarray technologies (such as CGH arrays or SNP arrays), but the better availability of whole genome sequencing at decreasing costs provides an analysis tool for an easily accessible combined one-step evaluation for CNV and monogenic disease genes in the near future. A CNV can identify a diagnostically relevant genetic cause of CP either in itself as a contiguous gene deletion/duplication syndrome (e.g., 22q11.2) or by identifying a monogenic disease through a deletion/duplication in a listed CP-gene. Oskoui et al. (18) identified *de novo* CNVs in 7% of their unselected Canadian CP cohort of 115 patients. Overall, 9.6% of the families carried clinically relevant CNVs either explaining etiology of CP or possibly accounting for associated medical complication. Another study detected abnormalities in 7.2% of patients with hemiplegic CP (19). Segel et al. found *de novo* CNVs in 31% of their cohort of Israeli patients with CP of unknown etiology (20). The most recent study reported by Corbett et al. (21) revealed pathogenic and likely pathogenic CNVs in 3.7% of their unselected CP cohort of 186 patients, using exome analysis. In summary, depending on the cohort, a pathogenic or likely pathogenic CNV is found in about 4–30% of CP patients (22).
- **Genetic sequence alteration in genes with a listed CP-related phenotype (Single gene mutations)**. To date, a large number of genes have been identified as associated to a defined clinical phenotype compatible with CP. This includes genes causing brain malformations such as lissencephalies or polymicrogyrias, as well as genes coding for specific ion channels, which have been related to ataxic CP [ITPR1, KCNC3, and SPTBN2 (15, 23)], genes for inborn errors of metabolism, which may mimic CP (24), and many other genes, e.g., KANK1 [role in actin polymerization, related to a spastic CP phenotype, (25)], adaptor protein 4 complex (26), ADD3 (15), GAD1 [catalyzes the conversion of gamma-aminobutyric acid, a major neurotransmitter (26)], just to name a selection.
  Genetic analyses performed in CP cohorts showed a monogenic cause in ~5% (27) to 15% (22) of cases, vastly differing between different subgroups. This factor may increase during the next years when comprehensive genetic analysis such as whole-genome sequencing (WGS) will be more broadly available and more CP genes presumably identified.

#### Genetic Research Studies

- **Single nucleotide polymorphisms (SNPs**) are non-rare specific genomic nucleotide exchanges. In contrast to the rare pathogenic sequence changes in monogenic disease genes, SNPs usually occur in a proportion of at least 1% in the general population. SNPs are often investigated in association studies, which are expected to provide indicators of predisposing factors for certain diseases and shed further light on the pathophysiology. In rare diseases, SNPs are not expected to have a sole causal effect but to act as vulnerability factors. They may gain further significance in the context of Polygenic Risk Score Assessments.
  In different studies, SNPs with susceptibility to thrombosis or hemorrhage have been suggested. However, a strong association to CP has not been found (15, 28). Apoprotein E is one of the most studied presumed risk factors. Whereas, the ApoE4 allele was associated with a severe clinical phenotype in some studies, other ApoE alleles were associated with reduced severity of CP (29). Larger studies found no association between ApoE and the risk of CP (30). The association between mutations in genes causing hereditary thrombophilia (antithrombin, Protein C, and Protein S) and CP due to perinatal stroke was weak (31).
- **Candidate cerebral palsy genes** Candidate genes are presumed to be monogenic disease genes for CP, but a verification of the causal effect by functional analyses is still missing. Candidate genes are usually identified in comprehensive genetic studies because they show rare sequence alterations [mostly in whole-exome sequencing (WES) or WGS] or located within a CNV (mostly in WGS or microarray). McMichael et al. (32) found novel candidate CP genes in 7% of an unselected CP cohort (183 cases) using WES.

### Role of Genetic Analysis in the Different Brain Pathology Patterns

#### Maldevelopments

Maldevelopments occur early during brain development and are often due to gene abnormalities, which are relevant in specific periods of early brain development. Maldevelopments of cortical development are here of specific importance. They often lead to spastic CP, depending on their localization and extent. Neuronal proliferation, migration, and organization or post-migrational development depend on a complexity of genetic mechanisms, which are increasingly recognized. Accordingly, mutations of many genes have been described in patients with malformations of cortical development (33). A comprehensive discussion of genetic causes is beyond the scope of this paper, but some classical examples are mentioned and illustrate that the origin can be genetic or non-genetic:

- *The lissencephalies* (migration disorders) include agyria, pachygyria, and subcortical band heterotopia as part of a spectrum. Associated gene defects include the LIS1, DCX, RELN, ARX, or TUBA1 genes (33–35). Bilateral spastic CP is usually severe and accompanied by severe cognitive impairment, epilepsy, and cortical visual impairment, and children are microcephalic.
- *Proliferation defects* associated to megalencephaly may also cause severe bilateral spastic CP and can occur due to *de novo* germline and postmitotic mutations in AKT3, PIK3R2, and PIK3CA (36). The latter has also been reported with hemimegalencephaly and unilateral spastic CP (37).
- *Polymicrogyrias* are defects of organization or post-migrational development and are characterized by the appearance of an excessive number of small cortical folds. Although a number of genes have been associated, a larger part seems still unclear with respect to the underlying mechanism (38). A clear genetic origin is given when polymicrogyria occurs in conjunction with early manifesting inborn errors of metabolism such as peroxisomal disorders or mitochondrial disorders (33). Then the clinical picture is not one of a CP, and MRI in addition may disclose other abnormalities suggesting a progressive disease. The origin of polymicrogyria is probably more often disruptive than genetic, for example, due to vascular events causing a schizencephalic pattern with clefts lined by polymicrogyric cortex (33). Additional calcifications and white matter abnormalities point toward an infectious origin such as cytomegalovirus (39). A pure symmetrical polymicrogyria may have a genetic background such as bilateral fronto-parietal polymicrogyria also called “frontal predominant cobblestone malformation,” which can occur due to GPR56 mutations.
- *Focal cortical dysplasias* (FCDs) are probably mainly due to “abnormal post-migrational development”. Evidence suggests that they can result from injury to the cortex during later stages of cortical development (33). Prenatal and perinatal insults including severe prematurity, bleeding, hydrocephalus, stroke, and others are reported in children with mild malformation of cortical development (40, 41).

#### Lesional Patterns (Predominant White or Gray Matter Injury)

As introduced above, research in CP has partly focused on genetic association studies, and an intriguing aspect is that genetically defined vulnerability factors can lead to neuroimaging abnormalities categorized as predominantly white matter or gray matter injuries (15), just as a genetic predisposition to stroke has long been recognized. However, studies have revealed no or only a small association between CP and the assumed genetic risk factors including mutations in genes causing hereditary thrombophilia.

A special role of the COL4A1 gene as a risk factor for lesional injuries has emerged in this context, which should be specially mentioned. Defects in the COL4A1 gene have been reported not only to increase the risk for stroke but also to occur in patients with prenatal brain lesions such as porencephaly and schizencephaly (42), accounting for 16 and 50% each in the latter series. Defects in the COL4A1 gene can be inherited (autosomal dominant with reduced penetrance) or occur *de novo*. As the phenotype mainly consists of a small (or large) vessel disease, which may involve the eyes and most commonly also other organs such as the kidneys, a thorough family assessment is important (43).

#### Miscellaneous Findings

The group “miscellaneous” refers to abnormal brain imaging findings, which cannot be allocated to the categories A, B, or C given above. SCPE registers are encouraged not only to code a finding but also describe it and give the MRI report. In its recent report, SCPE identified 8.5% of MRI findings as miscellaneous (11). For the purpose of this paper, we analyzed this group in more detail according to the additional descriptions given by the centers asking which findings could indicate a genetic cause. Findings were reported by SCPE registers from 18 European countries for children born between 1999 and 2009. Out of 3,818 MRI descriptions or written reports, 323 were coded as miscellaneous. No description was given for 22 cases (6.8 %).

Four groups of patterns could be characterized:

1. *Acquired patterns such as infections, tumor, or hemorrhage* represented 4.3% (14 cases) and included among others: prenatal infections (CMV, *n* = 3), encephalitis (Herpes, *n* = 5), tumor (*n* = 2), hemorrhage not covered by B or C (subdural/subarachnoidal hemorrhage).
2. *Brain injuries* that could not be classified into B or C as descriptions were too unspecific, such as “hypoxic injury,” “widespread injury,” represented 18.8% (61 cases).
3. Patterns which were *suggestive of a genetic background* accounted for 31.8% (103 cases): 83 cerebral/cerebellar atrophy and 20 myelination disorders (hypo- or demyelination).
4. *Unspecific patterns* such as thin corpus callosum, ventricular dilatation, arachnoid cysts, calcifications not specified as in context of a CMV-Infection, and other unspecific descriptions accounted for another 38% of this group (123 cases).

In groups 3 and 4, representing 70% of the miscellaneous group, genetic work-up certainly is to be recommended as further diagnostic procedure. Ideally, this should be done as WES with additional microarray analysis or WGS, as findings usually did not clearly indicate a specific monogenic disease.

#### Normal Findings

**Figure 1:**
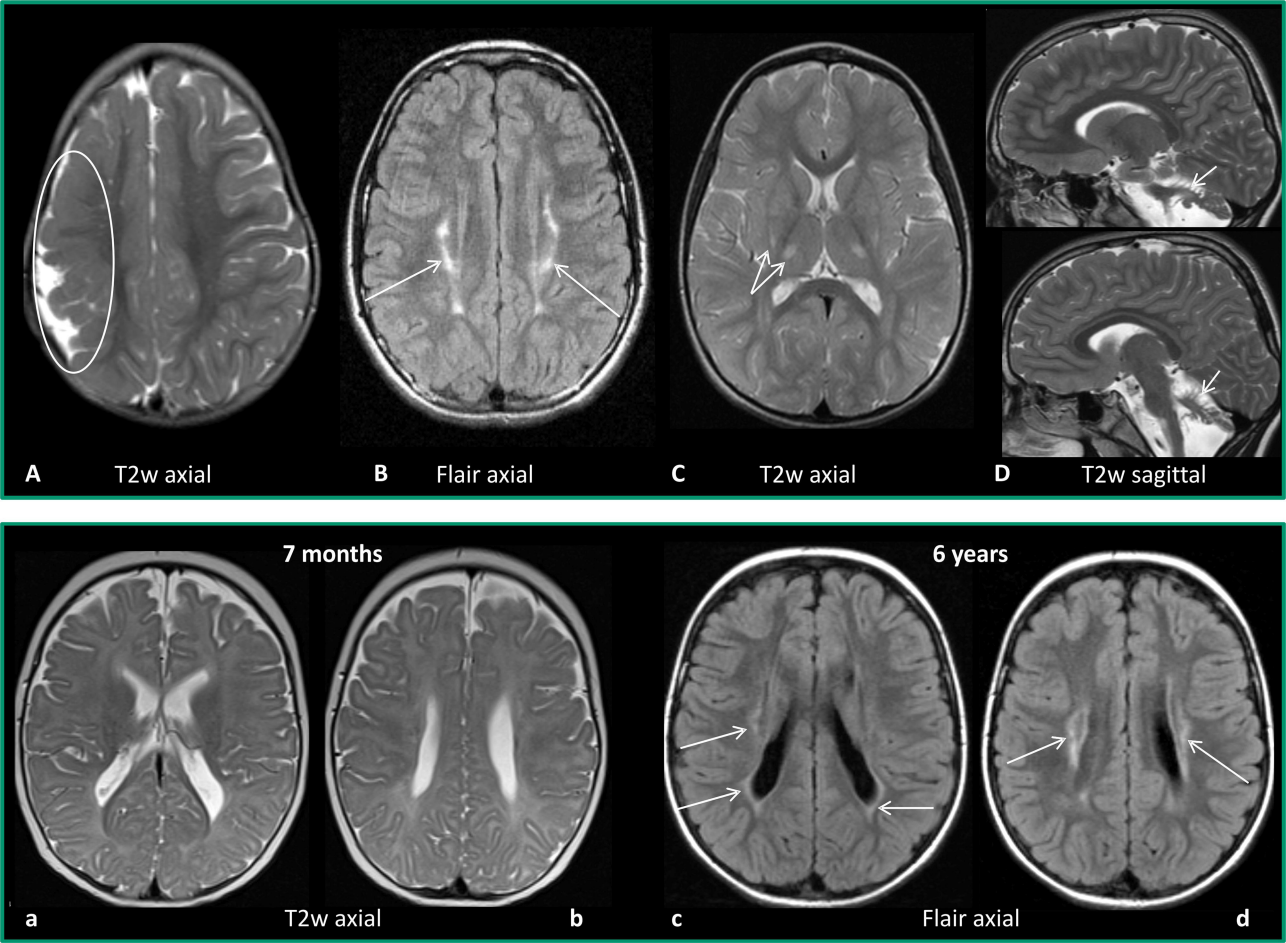
Upper row: Examples for MRI patterns. **(A)** Maldevelopment: frontoparietal polymicrogyria right (encircled), 2 years old girl with US-CP left GMFCS I. **(B)** Predominant white matter injury: mild PVL (arrows), 12-year-old girl, preterm 32 weeks of gestation, BS-CP GMFCS I. **(C)** Predominant gray matter injury: mild basalganglia/thalamus lesions (double arrow), 3-year-old girl, placental ablation, dyskinetic CP GMFCS II. **(D)** Miscellaneous: cerebellar atrophy (arrows), 2-year-old boy, former preterm 26 weeks of gestation, ataxic CP, GMFCS I. Lower row: Example for difficulty of early diagnosis–PVL, former preterm, 32 weeks of gestation. **(a,b)** MRI at the age of 7 months: T2w axial images with no clear pathology. **(c,d)** MRI at the age of 6 years (BSCP, GMFCS III): axial FLAIR images reveal bilateral gliosis indicating PVL (arrows).

Normal MRI findings accounted for about 10.5% of CP cases in the SCPE report. It is of essential importance, as said before, to verify and re-consider whether diagnosis of CP is correct. A first pitfall could be the age at MRI. Mild periventricular leukomalacia (PVL) without reduction of white matter may not be evident in an MRI done before myelination is complete (see Figure 1). Mild basal ganglia and thalamus lesions appear in the neonatal period hyperintense on T1w images, after the first year they are reported as hyperintense on T2w images; in the transition phase, especially small lesions may be missed. Thus, in a child with mild spastic or dyskinetic CP and no major cognitive problems, a normal MRI before the age of 2 years should be repeated when myelination is complete.

Spinal origin of spasticity in a young child may be difficult to differentiate clinically from cerebral origin. In a child with spasticity of the legs and no comorbidities, a spinal MRI should be considered if cerebral MRI is normal, not to miss structural spinal pathology.

The next step then is genetic testing. Two disease entities may mimic especially spastic CP. Although progressive, they may start early with very slow progression. Hereditary spastic paraplegias (HSPs) are clinically and genetically heterogeneous. When starting early, the course is often very slowly progressive (44), MRI is usually normal in pure HSP, and bladder dysfunction may not be present. SPG3 is probably the genotype most often associated with these features (45, 46). However, comprehensive genetic testing is suggested as the majority of genetic subtypes, age of onset, and phenotypic expression are extremely broad (44). Dopa-responsive dystonias (DRDs) are the second entity, which should be considered in a child with mild spastic (or dyskinetic) CP, normal cognition, and normal MRI. As diagnosis has therapeutical consequences, this should be done early. DRDs typically manifest as limb-onset, diurnally fluctuating dystonia. Autosomal dominant GTP cyclohydrolase 1 deficiency, also known as Segawa disease, is the most common and best-characterized condition (47). At disease onset, diurnal fluctuation may not yet be obvious, and with early onset, the neurological condition may be misdiagnosed as leg dominated bilateral spastic CP (48). As DRDs exhibit a rapid and clear response to levodopa treatment, a levodopa trial (mean 200 mg/day) may be used as the first diagnostic step, especially if genetic testing and cerebrospinal fluid neurotransmitter analysis are not easily available.

In ataxic CP presenting with normal MRI and normal cognition, some biochemical tests may be done before initiating broad genetic testing: serum AFP and ß-galactosidase as an indicator for Louis–Bar syndrome and GM1-gangliosidosis with late infantile onset, respectively.

A child with clinical signs compatible with CP of any subtype, a normal MRI but clear cognitive deficits always needs WGS or WES with microarray as a next diagnostic step.

## Concluding Discussion

Genetic studies in children with CP often have the shortcoming that phenotypic definition is not very strict, and usually, no or little information is given on brain pathology. We have discussed genetic findings reported in CP with respect to the underlying brain pathology according to neuroimaging findings, based on the distribution of MRI findings from an analysis of children with CP phenotypically defined according to SCPE guidelines (11). Brain pathology is classified according to a specific timing of the brain compromise. With a view to these groups, the role of genetic contribution to the causation of CP can be discussed more specifically, summarized also in Table 1.

**Table 1:** Recommendation for genetic work-up according to neuroimaging findings.

**Pattern**	**Genetic work up recommended**
A. Maldevelopments	**For monogenic diseases** “rule of thumb”: disorders of cortical formation proliferation and migration defects are of genetic origin, whereas later defects such as organization or post-migrational defects (polymicrogyria) can be of genetic or non-genetic origin
B. Predominant white matter injuries C. Predominant gray matter injuries	**For genetic vulnerability factors** such as defects leading to thrombophilia or interfere with collagen formation, e.g., some collagenopathies. Contribution is low, thus, check family history, and whether causative external factors are missing
D. Miscellaneous	**For monogenic diseases** indicators are, e.g., disorders of myelination (such as hypomyelination) or cerebral atrophy, cerebellar atrophy
E. Normal	**For monogenic diseases**
	before initiating genetic testing check on a child with good cognition and mild spastic or dyskinetic CP - has MRI been done before the age of 2 yrs? - has spinal MRI been done? - consider a levodopa trial

The largest groups are injuries predominantly of white or gray matter, accounting together for 70% of cases. Here genetic vulnerability factors may play a role. Up to now, evidence for their importance is not high. However, this needs further evaluation. If future studies on the genetic contribution to CP took up not only a more stringent definition of CP but also neuroimaging findings, results could be more conclusive, as indicated by the study centering on prenatal brain lesions, where a high proportion of COL41A defects could be identified, accounting for 16% of porencephalies and 50% of schizencephalies (42).

Malformations of the brain were reported in around 11% of children with CP. Here, cortical malformations play a specific role. As a “rule of thumb,” malformations characterizing early disturbance of brain development such as proliferation and migration defects are mostly of genetic origin, whereas later defects such as organization or post-migrational defects have a higher probability for a non-genetic origin.

Miscellaneous findings are an intriguing group of findings. With higher quality of reporting (a process of regular training and feedback is ongoing in SCPE), the 20% of brain injuries coded in this category will hopefully be more specifically characterized in the future. For the other findings, which cannot clearly be allocated to an acquired condition, a genetic work-up is to be recommended. These findings also point out that diagnosis of CP has to be questioned in this group, as MRI findings do not clearly indicate a static brain disorder as the cause of the CP. SCPE considers an age of 4 years necessary for inclusion of a child as having CP allowing for a certain observation period to make it unlikely that a progressive disorder is misdiagnosed as CP. In times of readily available genetic testing, it is, however, advisable to already initiate a genetic work-up before, if MRI is not clearly indicating an acquired injury. This holds true also for the last group of MRI pattern, the normal findings. Here, in addition, quality and timing of the MRI has to be checked, as small lesions of white matter or diencephalon may go unrecognized before myelination is complete.

Taken together, the known contribution of genetic findings to the understanding of CP according to the literature is <30%. In addition, when considering the MRI findings reported from the SCPE database, it seems interesting that around 30% cannot be allocated to an acquired lesion; thus, a genetic origin has to be considered (maldevelopments, miscellaneous, and normal findings).

Hopefully, future genetic studies will be able to give more specific results while relying not only on a standardized phenotypical description of children with CP but also on their neuroimaging findings characterizing brain pathology. Polygenic risk scores are emerging approaches which will, hopefully, shed more light on the role of genetic vulnerability factors. In the meantime, we suggest some recommendations when to do a genetic work-up in a child with CP based on neuroimaging, which we do consider as the first diagnostic step after medical history taking and neurological examination. This may also have implications for molecularly informed treatment decisions, as neuroimaging findings may support early genetic work-up in children with slowly progressive and treatable disorders mimicking CP (24, 47). Against the background of the current data situation and decreasing examination costs, the application of comprehensive analysis methods such as exome analyses and genome analyses is preferable today to the targeted use of special gene panels or single gene analyses, provided that these are accessible. This applies both to a diagnostic framework and to research settings.

## Data Availability Statement

The raw data supporting the conclusions of this article will be made available by the authors, without undue reservation.

## Author Contributions

VH: designed the manuscript, performed literature review and analyzed miscellaneous MRI data, drafted manuscript and performed revisions. UG: wrote section Genetic testing in CP, provided genetic expertise and reviewed the manuscript. ES: provided MRI data and expertise for MRI data analysis, reviewed the manuscript. CA: reviewed the manuscript. IK-M: conceived, designed and supervised the study, provided clinical expertise, participated in manuscript drafting and revision. KH: provided clinical expertise, supervised and revised the manuscript. All authors contributed to the article and approved the submitted version.

## Conflict of Interest

The authors declare that the research was conducted in the absence of any commercial or financial relationships that could be construed as a potential conflict of interest.
